# Longitudinal Analysis of Dengue Virus–Specific Memory T Cell Responses and Their Association With Clinical Outcome in Subsequent DENV Infection

**DOI:** 10.3389/fimmu.2021.710300

**Published:** 2021-07-28

**Authors:** Luis Alberto Sanchez-Vargas, Kathryn B. Anderson, Anon Srikiatkhachorn, Jeffrey R. Currier, Heather Friberg, Timothy P. Endy, Stefan Fernandez, Anuja Mathew, Alan L. Rothman

**Affiliations:** ^1^Department of Cell and Molecular Biology, Institute for Immunology and Informatics, University of Rhode Island, Providence, RI, United States; ^2^Department of Medicine, Department of Microbiology and Immunology, Institute for Global Health and Translational Sciences, State University of New York-Upstate Medical University, Syracuse, NY, United States; ^3^Faculty of Medicine, King Mongkut’s Institute of Technology Ladkrabang, Bangkok, Thailand; ^4^Viral Diseases Branch, Walter Reed Army Institute of Research, Silver Spring, MD, United States; ^5^Department of Virology, Armed Forces Research Institute of Medical Sciences, Bangkok, Thailand

**Keywords:** dengue (DENV), memory T cell analysis, cytokine - immunological terms, cultured ELISpot assays, interferon-gamma (IFN) and tumor necrosis factor-alpha (TNF)

## Abstract

Memory T cells resulting from primary dengue virus (DENV) infection are hypothesized to influence the clinical outcome of subsequent DENV infection. However, the few studies involving prospectively collected blood samples have found weak and inconsistent associations with outcome and variable temporal trends in DENV-specific memory T cell responses between subjects. This study used both *ex-vivo* and cultured ELISPOT assays to further evaluate the associations between DENV serotype-cross-reactive memory T cells and severity of secondary infection. Using *ex-vivo* ELISPOT assays, frequencies of memory T cells secreting IFN-γ in response to DENV structural and non-structural peptide pools were low in PBMC from multiple time points prior to symptomatic secondary DENV infection and showed a variable response to infection. There were no differences in responses between subjects who were not hospitalized (NH, n=6) and those who were hospitalized with dengue hemorrhagic fever (hDHF, n=4). In contrast, responses in cultured ELISPOT assays were more reliably detectable prior to secondary infection and showed more consistent increases after infection. Responses in cultured ELISPOT assays were higher in individuals with hDHF (n=8) compared to NH (n=9) individuals before the secondary infection, with no difference between these groups after infection. These data demonstrate an association of pre-existing DENV-specific memory responses with the severity of illness in subsequent DENV infection, and suggest that frequencies of DENV-reactive T cells measured after short-term culture may be of particular importance for assessing the risk for more severe dengue disease.

## Introduction

Dengue is one of the most important arboviral disease of humans, with half of the population at risk of infection (1). Dengue is caused by any of the four closely related dengue virus (DENV) serotypes belonging to the flavivirus family (2, 3). DENV infection most commonly results in inapparent infection but may manifest as dengue fever (DF) or dengue hemorrhagic fever (DHF) (4). Over 90% of severe cases occur during a secondary infection and it is well recognized that prior immunity constitutes one of the strongest risk factors for severe disease. The mechanism(s) underlying this risk remain a point of debate (5, 6).

Memory T cells from a primary DENV infection can be reactivated during secondary infection with peptides different from the prior DENV serotype and induced to express an altered profile of effector functions (7, 8). In studies of acute DENV infection, we and others have found very high frequencies of activated antigen-specific T cells in the peripheral blood during the acute phase and in early convalescence (9–14). However, 1-3 years post-infection, T cell responses to DENV have been low and in many cases undetectable. We recently reported T and B cell dynamics over five years in children from Thailand who were followed for the occurrence of DENV infections. We found low and fluctuating T cell responses in PBMC from children prior to infection in response to overlapping peptide pools that encompassed both structural and non-structural proteins using an *ex-vivo* ELISPOT assay (15).

Effector memory virus-specific T cells are a subpopulation of memory T cells capable of secreting cytokines such as IFN-γ when stimulated with their cognate MHC-peptide, a characteristic used for their *ex-vivo* detection in ELISPOT assays (16). Cultured ELISPOT assays, in contrast, leverage short-term (days to weeks) *in vitro* culture of memory T cells principally to increase assay sensitivity, but some data suggest that this method predominantly detects central memory T cells that need restimulation to elicit effector function (16). Cultured ELISPOT assays have been used to detect memory T cell responses to DENV peptides in people with mild and subclinical infection (17, 18).

In this study, we sought to define the associations between T cell responses in school age subjects enrolled in a prospective dengue cohort before secondary infection and the clinical severity outcome of that secondary infection. In consideration of the above issues, we studied memory T cell responses prior to and following symptomatic secondary infection using both *ex-vivo* and cultured ELISPOT assays. We found higher frequencies of cytokine-secreting T cells in response to stimulation with DENV peptides using the cultured ELISPOT assay, and found a significant association with the occurrence of DHF during a subsequent DENV infection.

## Materials And Methods

### Study Subjects

Subjects were selected from a 5-year prospective cohort study of schoolchildren in Kamphaeng Phet province, Thailand, previously described (19). Blood samples were collected in January and February each year. Active surveillance for febrile illnesses was conducted between June and November to detect incident symptomatic DENV infections, and these were classified based on whether they were hospitalized (a decision made by the treating medical staff) and whether they fulfilled WHO criteria for DHF (20). For the present study, we identified participants who had experienced a symptomatic, laboratory-confirmed secondary DENV infection during 2001 and were classified as either non-hospitalized DF (NH) or hospitalized DHF (hDHF). We further selected subjects who had tested positive by RT-PCR for DENV2 or DENV3 and who had cryopreserved PBMC samples available at least from the annual blood draws prior to and after the detected infection. Primary or secondary DENV infection were distinguished by IgM/IgG ratio and hemagglutination inhibition assay titers as described elsewhere (19, 21).

Written informed consent was obtained from each subject or his/her parent or guardian. This study was approved by the Institutional Review Board of the Thailand Ministry of Public Health, the Human Use Review and Regulatory Agency of the Office of the U.S Army Surgeon General and the Institutional Review Board of the University of Massachusetts School of Medicine.

### Peptide Pools

Four overlapping peptide pools spanning the prM and E proteins from the four DENV types (designated 1prM/E, 2prM/E, 3prM/E, 4prM/E), four peptide pools spanning the non-structural proteins NS1, NS3 and NS5 from the four DENV types (designated 1NSA, 2NSA, 3NSA, 4NSA) and one peptide pool spanning C, NS2A/B, and NS4A/B proteins of DENV2 (designated 2NSB) were used to stimulate T cells. Peptides were obtained from the NIH Biodefense & Emerging Infections Research Resources Repository (BEI Resources, Bethesda, MD, USA) and Peptide technologies (JPT, Acton, MA, USA), as previously described (15). Peptides ranged in length from 12 to 20 amino acids and sequential peptides overlapped by 10 to 14 amino acids.

### *Ex Vivo* Single Color IFN-γ ELISPOT Assay

The *ex-vivo* ELISPOT assay was performed according to the manufacturer’s instructions using the reagents for IFN-γ detection from the Human TNF-α/IFN-γ double-color enzymatic ELISPOT assay kit (hIFNgTNF-a-1M/10, CTL, Cleveland, OH, USA; **Figure S1**). Briefly, ELISPOT assay plates were coated overnight with the IFN-γ capture antibody only. Cryopreserved PBMC were thawed and plated at a density of 1 x 10^5^ cells/well in a final volume of 200 µl. Peptide pools were added at a final concentration of 2 µg/ml/peptide. As a positive control, PBMC were incubated with anti-CD3 and anti-CD28 antibodies at final concentrations of 1 and 0.1 µg/ml, respectively. As a negative control, PBMC were incubated with medium alone. PBMC were incubated for 45 hr at 37°C with 5% CO_2_. The number of spots per well was determined using an automated ELISPOT reader (S5UV analyzer, CTL, Cleveland, OH, USA) with the single color software. Determinations from duplicate wells were averaged. Data were analyzed by subtracting the mean number of spots in the wells with cells and medium-only from the mean counts of spots in wells with cells and antigen and expressed as spot-forming cells (SFC) per 10^6^ PBMC.

### Cultured TNF-α/IFN-γ Dual-Color ELISPOT Assay *In Vitro*

Cultured ELISPOT was performed as previously described (22) with some modifications using the Human TNF-α/IFN-γ double-color enzymatic ELISPOT assay kit (hIFNgTNF-a-1M/10, CTL, Cleveland, OH, USA). **Figure S1** shows a schematic diagram of the cultured ELISPOT assay protocol. After thawing, 1-2 x 10^6^ PBMC/1ml/well in 48 well flat-bottom plates were stimulated with 1μg/ml of DENV peptide pools (15) for 12 days at 37°C ± 5% CO_2_. IL-2 (100 U/ml) was added on days 2 and 7. Non-structural peptide pools were prioritized over structural pools if there were not enough PBMC. On day 12, the cells were harvested and rested for an additional two days in RPMI-1640 supplemented with L-glutamine, 25 mM HEPES, 10% FBS and 1% penicillin/streptomycin. Cells were then evaluated *in vitro* using a dual-color ELISPOT for TNF-α/IFN-γ at 5 x 10^4^/well in duplicate and stimulated with DENV peptide pools (2μg/ml) of the secondary infecting serotype for 45 h at 37°C ± 5% CO_2_. If sufficient cells were recovered, DENV peptide pools from other serotypes were tested. Anti-CD3/CD28 was used as a positive control. The response was expressed as SFC/50,000 cells.

### Statistical Analysis

Statistical analysis was performed using GraphPad Prism software V 9.00 (GraphPad Software Inc., La Jolla, CA). The non-parametric Mann-Whitney test, Wilcoxon matched-pairs signed-rank test, and Kruskal-Wallis with Dunn’s multiple comparison post-test were used as appropriate. To determine associations between different variables, a two-tailed Spearman’s correlation test was used. Statistical significance was set at P < 0.05.

## Results

### Low Frequencies of DENV-Specific Memory T Cells Detected by *Ex Vivo* ELISPOT

**Figure 1:**
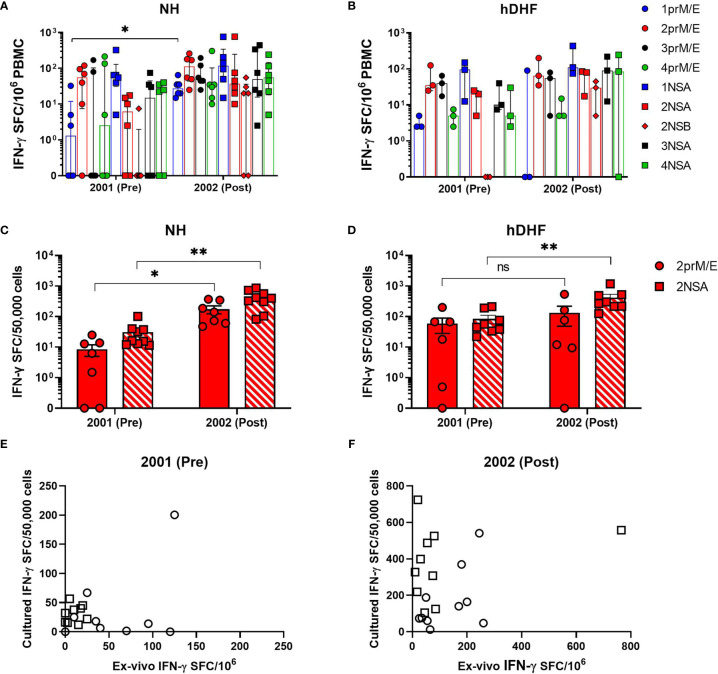
Comparison of *ex-vivo* and cultured IFN-γ ELISPOT. DENV-specific IFN-γ spot-forming cells (SFC) were determined by *ex-vivo* **(A, B)** and cultured ELISPOT **(C, D)** in PBMCs collected before (pre) and after (post) secondary DENV-2 infection in NH **(A, C)** and hDHF **(B, D)** subjects. PBMC were stimulated with peptide pools corresponding to prM and E proteins (prM/E), NS1, NS3, and NS5 proteins (NSA), or C, NS2A/B, and NS4A/B proteins (NSB) of the indicated DENV serotype. Bars represent median + interquartile range. Graphs **(E, F)** depict correlation analysis between frequencies of DENV-specific T cells detected by *ex-vivo* and cultured ELISPOT- 2001 2prM/E (r=0.07531, p= 0.8525); 2001 2NSA (r= 0.2343, p= 0.5397); 2002 2prM/E (r=0.2121, p= 0.5603); 2002 2NSA (r= 0.1152, p= 0.7589). Frequency of cytokine-producing cells in PBMC from each subject represented by circles (response to 2prM/E) and square (response to 2NSA). Statistics were calculated by the non-parametric Wilcoxon matched-pairs signed-rank test (**A–D**, comparing responses to the same peptide pool between 2001 and 2002) and two-tailed Spearman’s correlation test **(E, F)**. *p < 0.05, **p < 0.01, ns, not significant.

We first assessed IFN-γ responses to the four DENV serotypes in PBMCs collected before or after symptomatic secondary DENV2 (19 PBMCs from 10 patients) and DENV3 (8 PBMCs from 4 patients) infections using an *ex-vivo* ELISPOT assay (**Figure S2**). Characteristics of the study subjects are shown in **Table 1**. Based on our selection criteria, all subjects had their symptomatic secondary DENV infection during 2001. None of the subjects had an earlier DENV infection detected during their study observation period; therefore, no information is available on the timing or serotype of the earlier infection. Frequencies of memory T cells secreting IFN-γ in response to DENV peptide pools were low in most subjects before secondary DENV2 infection with no significant increases after infection in NH or hDHF subjects (**Figures 1A, B**). Similar trends in the IFN-γ responses were found in subjects with secondary DENV3 infections (**Figure S2C**). In addition, IFN-γ responses to DENV2 or DENV3 peptide pools were not dominant compared to the other serotypes before or after DENV2 or DENV3 infection, respectively (**Figure S2**).

**Table 1:** Characteristics of the study population.

Donor#	Year of enrollment	Age (y)^a^	Gender^b^	Dengue Serotype^c^	Clinical manifestation^d^	PBMC studied	*Ex-vivo* ELISPOT	Cultured ELISPOT
1	1998	12	M	DENV2	NH	1998, 1999^e^, 2000, 2001, 2002	✓	✓
2	1998	11	F	DENV2	NH	1998, 1999^e^, 2000, 2001, 2002	✓	✓
3	1998	15	F	DENV2	NH	1998, 1999^e^, 2000, 2001, 2002	✓	✓
4	1999	10	F	DENV2	NH	2001, 2002	✓	✓
5	1999	10	F	DENV2	NH	2001, 2002	✓	✓
6	1999	10	M	DENV2	NH	2001, 2002	✓	✓
7	1998	11	M	DENV2	NH	1998, 1999^e^, 2000, 2001, 2002^e^		✓
8	1998	12	F	DENV2	NH	1998, 1999^e^, 2000, 2001, 2002		✓
9	1999	10	M	DENV2	NH	2001^e^, 2002		✓
10	2001	10	M	DENV2	NH	2001^e^, 2002		✓
11	1999	10	M	DENV2	NH	2001^e^, 2002		✓
12	1998	11	F	DENV2	hDHF1	1998, 1999, 2000, 2001, 2002	✓	✓
13	1999	10	M	DENV2	hDHF1	2001, 2002	✓	✓
14	2001	8	F	DENV2	hDHF1	2001, 2002	✓	✓
15	1998	11	F	DENV2	hDHF1	1998, 1999^e^, 2000, 2001^e^, 2002		✓
16	1998	11	F	DENV2	hDHF1	1998^e^, 1999^e^, 2000, 2001, 2002^e^		✓
17	1998	12	M	DENV2	hDHF1	1998, 1999, 2000, 2001^e^, 2002		✓
18	2000	8	M	DENV2	hDHF1	2001, 2002		✓
19	2001	8	M	DENV2	hDHF2	2001, 2002	✓	✓
20	1999	10	F	DENV2	hDHF2	2001, 2002		✓
21	1998	12	M	DENV3	NH	1998, 1999, 2000, 2001, 2002	✓	✓
22	1998	13	M	DENV3	NH	1998, 1999^e^, 2000, 2001, 2002	✓	✓
23	1998	13	M	DENV3	NH	1998, 1999, 2000, 2001, 2002	✓	✓
24	1998	12	M	DENV3	NH	1998, 1999, 2000, 2001, 2002	✓	✓

a Age at the time of the secondary infection.

b F, female; M, male.

c All secondary infections were in 2001.

d NH, nonhospitalized; hDHF1, hospitalized dengue hemorrhagic fever grade I; hDHF2 hospitalized dengue hemorrhagic fever grade II [according to the World Health Organization (20)].

e Inadequate sample- no data or only partial data was available.

### *In Vitro* Stimulation Increases the Frequency of Cytokine-Secreting DENV-Specific Memory T Cells After Infection

**Figure 2:**
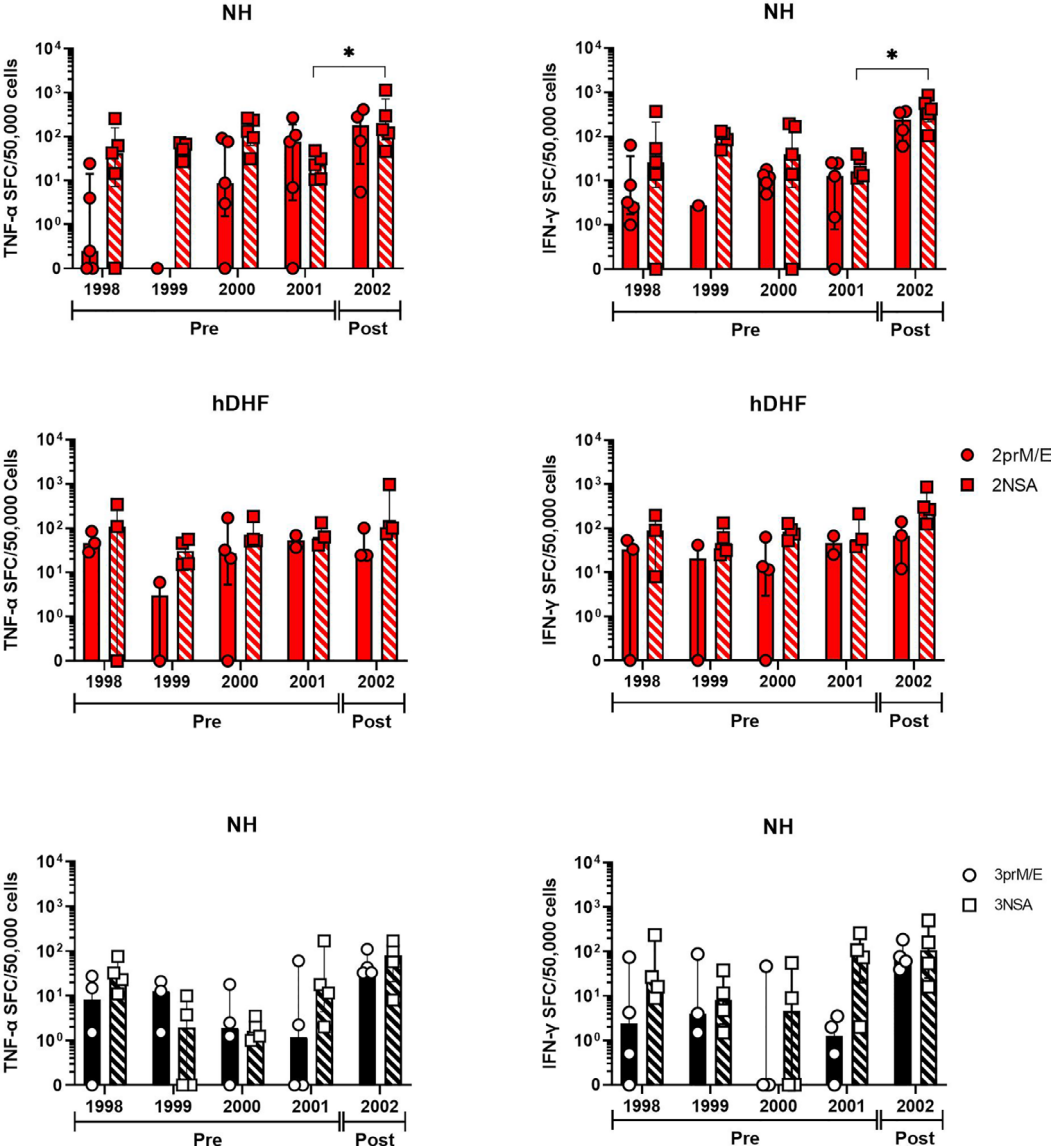
Trends in DENV-specific cytokine-producing T cell frequencies over the 5-year study period. Longitudinal analysis of cultured TNF-α (left panels) and IFN-γ (right panels) spot-forming cell (SFC) frequencies in PBMC collected before (pre) or after (post) secondary DENV infections in subjects with **(A)** DENV-2, non-hospitalized (NH) **(B)** DENV-2, hospitalized DHF (hDHF), and **(C)** DENV3 NH. Symbols represent the frequency of cytokine-producing cells in PBMC from each subject detected by cultured ELISPOT. PBMC were stimulated with peptide pools corresponding to prM and E proteins (prM/E) or NS1, NS3, and NS5 proteins (NSA) of the indicated DENV serotype. Bars represent median + interquartile range. Statistics were calculated by the nonparametric Kruskal-Wallis with Dunn’s multiple comparison post-tests. *p < 0.05.

The finding that low IFN-γ responses were detected by *ex-vivo* ELISPOT assays led us to evaluate responses using a more sensitive double color TNF-α and IFN-γ, cultured ELISPOT assay. T cell responses were studied in 83 PBMC samples from 24 subjects after 12 days of *in vitro* culture with DENV peptide pools (**Figures S3**, **S4**). Using this method, increased responses to DENV were clearly detectable in subjects following secondary DENV2 infection (**Figures 1C, D**) and DENV3 infections (**Figures 2** and **S3**). As shown in **Figures 1E, F**, no significant correlation was found between the IFN-γ producing cells detected by the *ex-vivo* compared to the cultured ELISPOT assay.

In a subset of these subjects, we were able to evaluate T cells secreting TNF-α or IFN-γ in response to DENV peptide pools in five sequential annual blood samples, four of which were collected before the secondary DENV infection. Responses in these earlier PBMC samples were generally similar in magnitude to the PBMC most proximal to the secondary infection (**Figure 2**), although a few subjects appeared to experience a decline in cultured ELISPOT responses that preceded the secondary infection (e.g., subjects 1, 15 and 24). PBMC from most subjects had a higher frequency of memory T cells secreting IFN-γ compared to TNF-α (**Figure S5**). Taken together, these results suggest that cultured ELISPOT was more sensitive to detect DENV-specific memory T cells compared to *ex-vivo* ELISPOT and different T cell populations are detected between the assays.

### DENV-Specific Memory T Cells Are Associated With the Severity of the Disease

**Figure 3:**
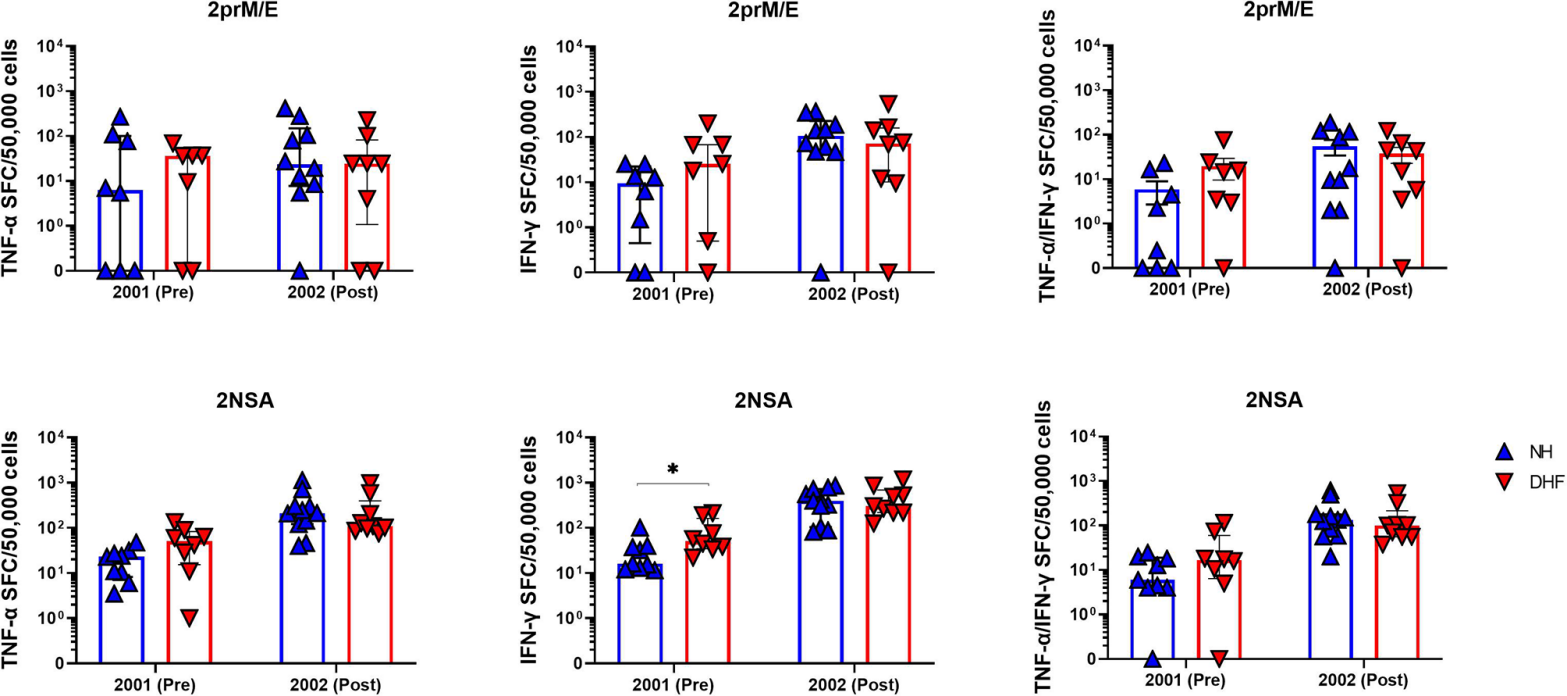
Higher DENV-specific T cell responses prior to secondary infection in hospitalized DHF subjects than non-hospitalized subjects. Symbols represent the frequencies of cells producing TNFα (left), IFNγ (middle), or both TNFα and IFNγ (right) by cultured ELISPOT in PBMC collected before (pre) or after (post) secondary DENV infection. PBMC were stimulated with peptide pools corresponding to DENV2 prM and E proteins (2prM/E, top) or DENV2 NS1, NS3, and NS5 proteins (2NSA, bottom). Bars represent median + interquartile range. Statistics were calculated by the non-parametric Mann-Whitney test. *p < 0.05.

We hypothesized that memory T cells resulting from primary DENV infection could associate with the clinical outcome of subsequent DENV infection. T cell responses to DENV peptides detected by *ex-vivo* ELISPOT in NH and hDHF subjects were not significantly different before or after secondary DENV infection. However, the frequency of IFN-γ-secreting DENV-specific memory T cells measured in the cultured ELISPOT assays was significantly higher in hDHF subjects than in NH subjects in the PBMC collected prior to secondary infection (**Figure 3**). Responses detected after secondary infection were similar between NH and hDHF subjects.

## Discussion

In this study, we measured DENV-specific memory T cell responses in sequential annual PBMC samples extending across the period prior to and after an acute febrile illness associated with viremic (RT-PCR-positive) secondary DENV infection. Consistent with a previous study in this cohort (15), *ex-vivo* ELISPOT assays using peptide pools representing all four DENV serotypes detected overall low frequencies of IFNγ-secreting cells prior to symptomatic secondary infection and inconsistent increases in these responses after infection. The present study builds on these findings by demonstrating the presence of low-level responses for multiple years before infection in most of the subjects tested. Further analysis using cultured ELISPOT assays more consistently detected pre-existing memory T cells specific for the DENV serotype which subsequently infected these individuals, and also reliably revealed a clear increase in the responses following secondary infection. Importantly, we found that PBMC from subjects who experienced hDHF during their secondary infection had significantly higher frequencies of IFN-γ-secreting cells prior to infection compared to those who experienced NH dengue; this observation was not evident using *ex-vivo* ELISPOT assays.

The more reliable detection of DENV-specific T cell responses using cultured ELISPOT assays as compared to *ex-vivo* ELISPOT assays and the poor correlation in magnitudes measured by the two assays are consistent with the findings of other groups (17, 22–24). An expansion in numbers of antigen-specific T cells is considered an important element of the enhanced sensitivity of cultured ELISPOT assays. Further, the observation that some memory T cells are unable to secrete sufficient quantities of cytokines to be detected in *ex-vivo* ELISPOT assays, yet are indeed activated by *in vitro* stimulation so as to be detected in cultured ELISPOT assays may be of equal or greater importance for explaining the differences between the results of these two methods (16). Secondary DENV infection is understood to involve a different serotype than the prior infection. Therefore, the memory T cell responses of interest to detect prior to a secondary infection will be responding (during the secondary infection) to peptides that are usually different in sequence from the antigen that had selected them, which we and others have shown can alter the response (7, 8, 25). The qualitative differences in responses detected by *ex-vivo* and cultured ELISPOT assays may therefore be particularly relevant in the context of DENV.

Frequencies of IFN-γ-secreting cells were overall higher than frequencies of TNF-α-secreting cells, and relatively few cells secreted both cytokines. This finding was somewhat unexpected, as we previously reported a bias towards TNF-α over IFN-γ production in response to stimulation of DENV-specific T cells with peptides of heterologous serotypes, and an association of secretion of TNF-α, but not IFN-γ, by pre-infection PBMC with hospitalization during secondary infection (7, 26). These differences might reflect the relative sensitivities of the assays used in each study, differences between production and secretion of TNF-α, localized secretion of TNF-α onto target cells, or some combination of these factors. Further functional studies of DENV-specific T cells might help to clarify these possibilities.

There is still considerable debate whether memory T cells play solely a protective role in secondary DENV infection or may instead have the potential to contribute to immunopathological mechanisms leading to plasma leakage/DHF (27). We previously proposed that the functions of virus-specific T cells allow them to have both effects, and that the collective quantity and quality of their responses determine the outcome of infection, as has been demonstrated in various models of virus infection, such as LCMV and vaccinia (28, 29). DENV-specific antibodies also are associated both with protection from infection and/or illness and to an increased risk of DHF (30), and can modify the interactions between T cells and antigen-presenting cells (31), further complicating the associations of each arm of adaptive immunity with outcome. It should be noted that all of the subjects included in this study experienced a symptomatic secondary DENV infection, meaning that their pre-existing DENV-specific immunological memory was insufficient to prevent viremia or illness. In our earlier study, we reported that cohort subjects who did not have a symptomatic infection showed higher responses to DENV peptides in *ex-vivo* ELISPOT assays (15). Therefore, the current finding that subjects who experienced DHF had higher pre-infection responses in cultured ELISPOT assays is consistent with this model of dual roles of pre-existing memory T cells in secondary DENV infections.

Our findings must be considered in the context of several important limitations of the study. Our study cohort was limited to primary schoolchildren living in a province of Thailand with high annual seroconversion rates. The T cell responses we observed in these annual blood samples might not be representative of cohorts of different ages or genetic backgrounds or with DENV exposures. Given the age of the study subjects, the quantity of PBMC available limited testing of responses to only a small number of antigens. In several of these subjects where sufficient PBMC were available, we did find higher responses in cultured ELISPOT assays to peptides from a different DENV serotype, perhaps representing the serotype of the earlier primary infection (data not shown); however, the fact that we measured responses to the serotype that was detected during the secondary infection should strengthen the importance of the association with outcome we observed. The limited quantity of PBMC also limits our ability to draw conclusions regarding the roles of other T cell effector functions in disease risk, the relative contribution of CD4 *versus* CD8 T cells to the observed associations, or alternative protocols for the cultured ELISPOT assay. These and other questions will need to be addressed in subsequent studies, unfortunately using PBMC samples from other individuals.

In conclusion, our data identify memory T cell responses measured in cultured ELISPOT assays using PBMC collected prior to secondary DENV infection that are associated with the clinical outcomes of DHF *versus* milder symptomatic illness. Immunological predictors of clinical outcome of DENV infection are in high demand to support the development and testing of vaccines against dengue. Our data suggest that cultured ELISPOT assays warrant further consideration and testing as part of that effort. It is possible that a combination of multiple assays will be necessary to discriminate the sometimes conflicting *in vivo* effects of cellular immunity.

## Data Availability Statement

The original contributions presented in the study are included in the article/**Supplementary Material**. Further inquiries can be directed to the corresponding author.

## Ethics Statement 

The studies involving human participants were reviewed and approved by Institutional Review Board, Ministry of Public Health, Thailand Human Use Review and Regulatory Agency, Office of the U.S Army Surgeon General Institutional Review Board, University of Massachusetts School of Medicine. Written informed consent to participate in this study was provided by the participants’ legal guardian/next of kin. Written informed consent was obtained from the minor(s)’ legal guardian/next of kin for the publication of any potentially identifiable images or data included in this article.

## Author Contributions

LS-V, AM, and AR conceived and designed the experiments and wrote the manuscript text. LS-V performed experiments and prepared figures. LS-V conducted statistical analyses. KA and TE supervised the clinical study, subject enrollment, and collection of clinical data and blood samples. All authors contributed to the analysis of clinical, virologic, and immunologic data. All authors contributed to the article and approved the submitted version.

## Funding

This work was supported by Program Project grant P01 AI034533 from the National Institutes of Health/National Institute of Allergy and Infectious Diseases and the U.S. Military Infectious Diseases Research Program, and utilized core facilities supported by NIH grant P20 GM104317.

## Author Disclaimer

Material has been reviewed by the Walter Reed Army Institute of Research. There is no objection to its presentation and/or publication. The opinions or assertions contained herein are the private views of the authors, and are not to be construed as official, or as reflecting true views of the National Institutes of Health, Department of the Army or the Department of Defense. The investigators have adhered to the policies for protection of human subjects as prescribed in AR 70–25.

## Conflict of Interest

The authors declare that the research was conducted in the absence of any commercial or financial relationships that could be construed as a potential conflict of interest.

## Publisher’s Note

All claims expressed in this article are solely those of the authors and do not necessarily represent those of their affiliated organizations, or those of the publisher, the editors and the reviewers. Any product that may be evaluated in this article, or claim that may be made by its manufacturer, is not guaranteed or endorsed by the publisher.
