# Supplementary material for: Longitudinal Analysis of Dengue Virus–Specific Memory T Cell Responses and Their Association With Clinical Outcome in Subsequent DENV Infection
Source: Front Immunol. 2021 Jul 28;12:710300. doi: 10.3389/fimmu.2021.710300 (PMC8355709; doi:10.3389/fimmu.2021.710300)
Supplement: Supplementary file 1 [file DataSheet_1.pdf]

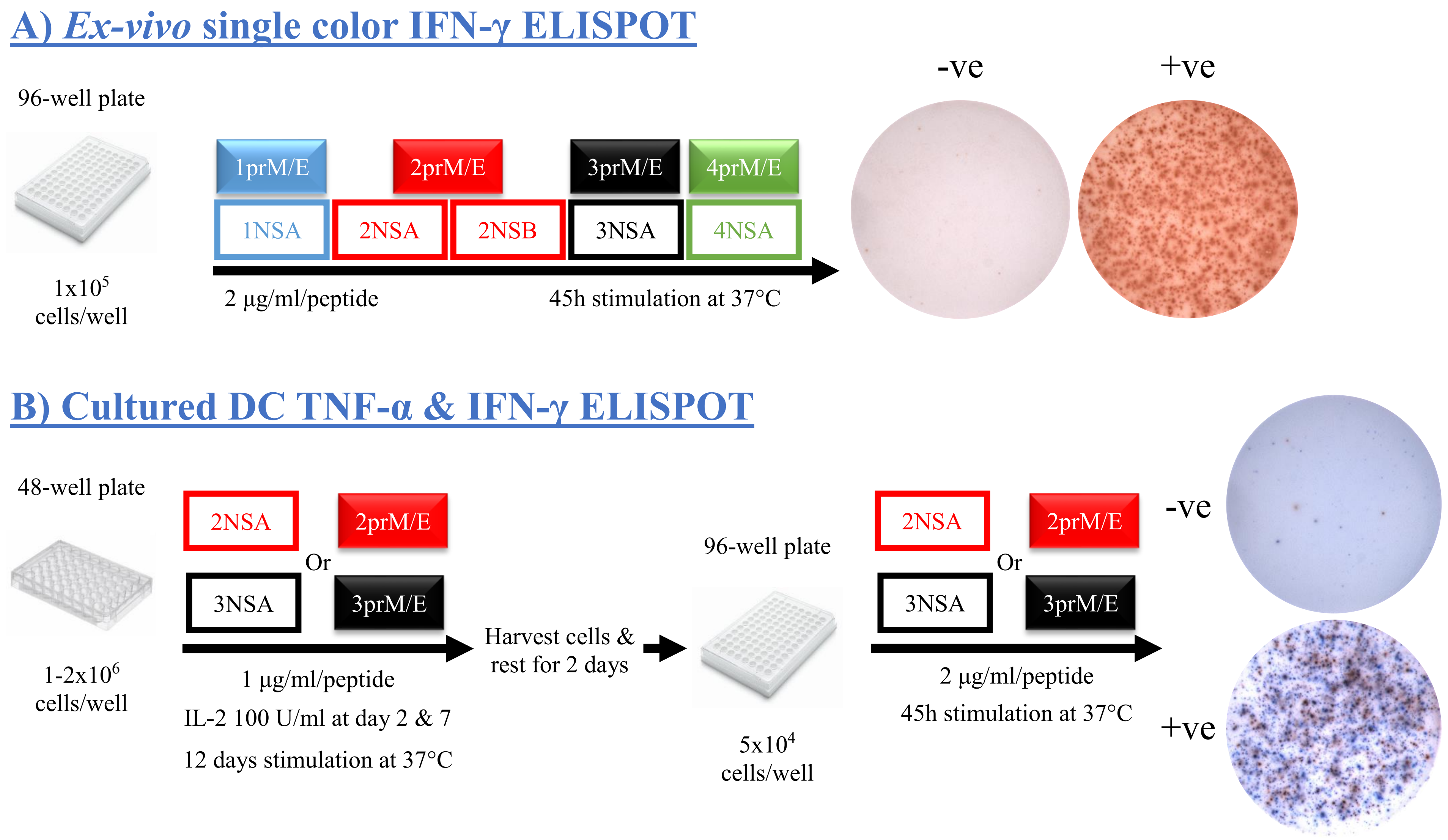
A) Ex-vivo single color IFN-γ ELISPOT
96-well plate
1x10^5 cells/well
1prM/E
2prM/E
3prM/E
4prM/E
1NSA
2NSA
2NSB
3NSA
4NSA
2 µg/ml/peptide
45h stimulation at 37°C
-ve
+ve
B) Cultured DC TNF-α & IFN-γ ELISPOT
48-well plate
1-2x10^6 cells/well
2NSA
2prM/E
Or
3NSA
3prM/E
1 µg/ml/peptide
IL-2 100 U/ml at day 2 & 7
12 days stimulation at 37°C
Harvest cells & rest for 2 days
96-well plate
5x10^4 cells/well
2NSA
2prM/E
Or
3NSA
3prM/E
2 µg/ml/peptide
45h stimulation at 37°C
-ve
+ve

C) Similar sensitivity of IFN-γ detection by *ex-vivo* and cultured ELISPOT

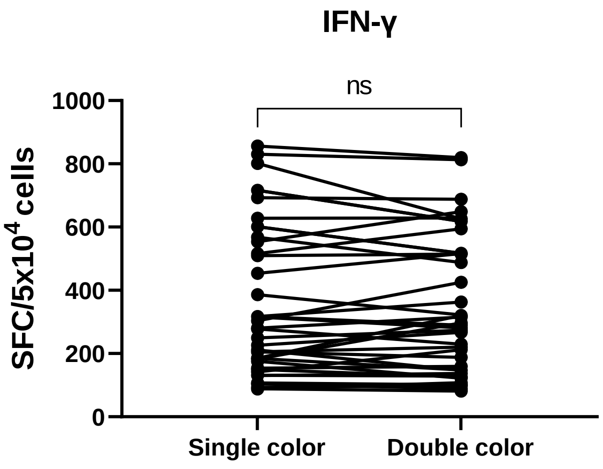

Figure S1. *Ex-vivo* and cultured ELISPOT procedures. A) Schematic diagram of *ex-vivo* ELISPOT. Cryopreserved PBMC were plated in a 96-well plate at a density of 1 x 10^5 cells/well. Peptide pools were added at a final concentration of 2 µg/ml/peptide. PBMC were incubated for 45 hr at 37°C with 5% CO_2. B) Schematic diagram of cultured ELISPOT. 1-2 x 10^6 PBMC/1ml/well in 48-well plate were stimulated with 1µg/ml of DENV peptide pools for 12 days at 37°C ± 5% CO_2. IL-2 (100 U/ml) was added on days 2 and 7. On day 12, the cells were harvest and rested in RPMI media for an additional two days. Cells were evaluated *in vitro* using a dual-color enzymatic ELISPOT for TNF-α/IFN-γ. Cultured cells were added at 5 x 10^4/well in duplicate and stimulated with DENV peptide pools (2µg/ml) of the secondary infecting serotype for 45 h at 37 °C ± 5% CO2. TNF-α spots are in blue, IFN-γ spots are in red and TNF-α/IFN-γ are in purple. C) IFN-γ responses measured using the reagents for IFN-γ (single color) or IFN-γ together with TNF-α (double color) in parallel on the same samples. There were no differences in the numbers of IFN-γ SFC when the reagents were only for IFN-γ detection compared to IFN-γ/TNF-α (Wilcoxon test, p=0.5077).

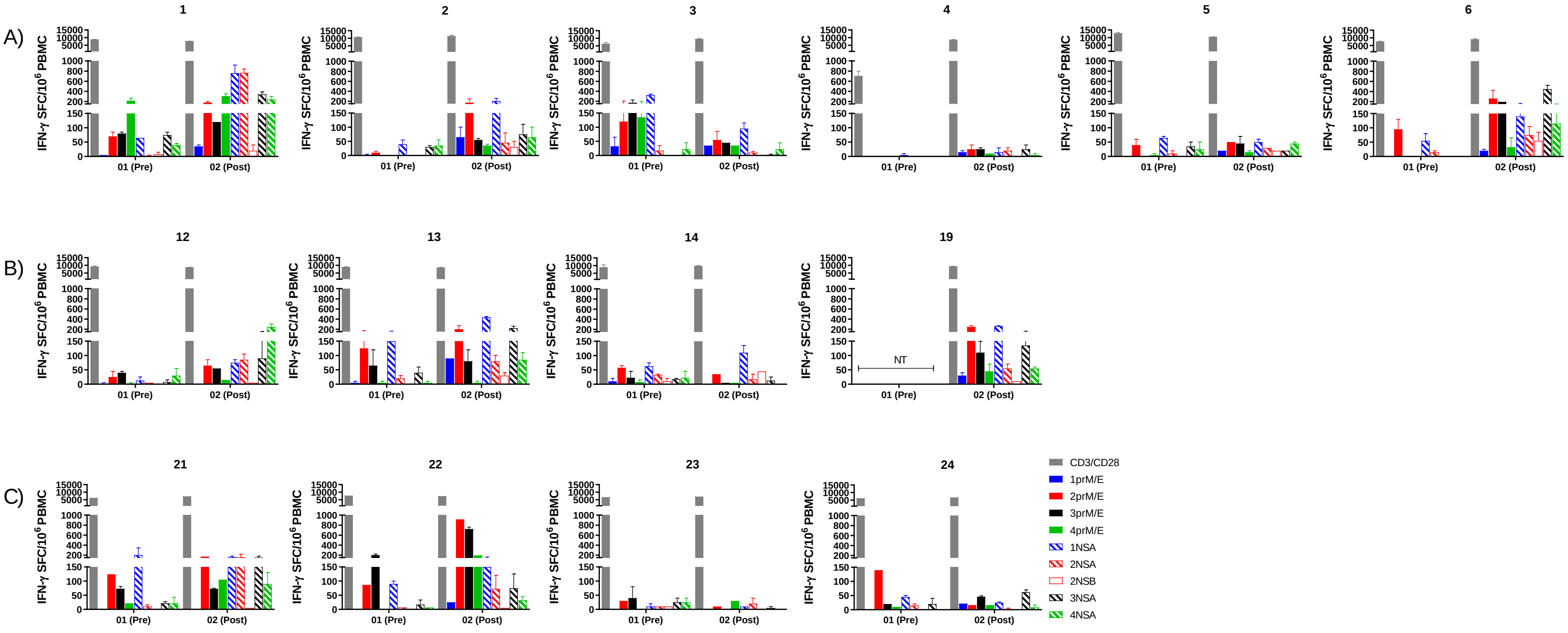

Figure S2. IFN-γ responses tested by *ex-vivo* ELISPOT in individual subjects before and after a secondary DENV infection. DENV peptide-specific interferon γ (IFN-γ) spot-forming cells (SFC) were determined by *ex-vivo* IFN-γ ELISPOT in PBMC collected before and after a secondary infection in A) DENV-2 NH B) DENV-2 hDHF and C) DENV3 NH. PBMC were stimulated with peptide pools corresponding to prM and E proteins (prM/E), NS1, NS3, and NS5 proteins (NSA), or C, NS2A/B, and NS4A/B proteins (NSB) of the indicated DENV serotype. Bars represent mean with the standard error of the mean from duplicate wells. NT, not tested.

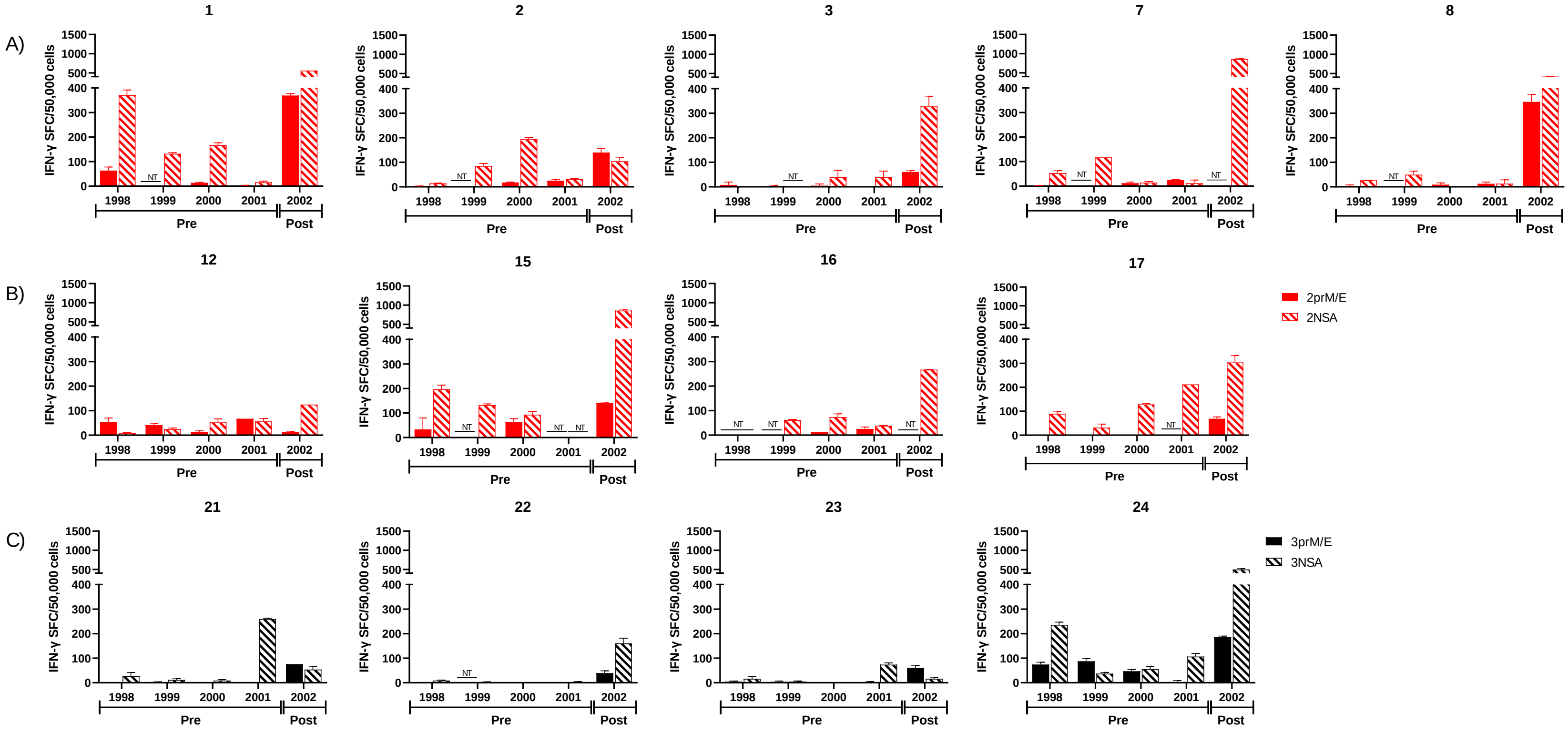

Figure S3. IFN-γ responses tested by cultured ELISPOT in individual subjects over the 5-years study period. DENV peptide-specific interferon γ (IFN-γ) spot-forming cells (SFC) were determined by cultured IFN-γ ELISPOT in PBMC collected during 5 years in A) DENV-2 NH B) DENV-2 hDHF and C) DENV3 NH. PBMC were stimulated with peptide pools corresponding to prM and E proteins (prM/E) or NS1, NS3, and NS5 proteins (NSA) of the indicated DENV serotype. Bars represent mean with the standard error of the mean from duplicate wells. NT, not tested.

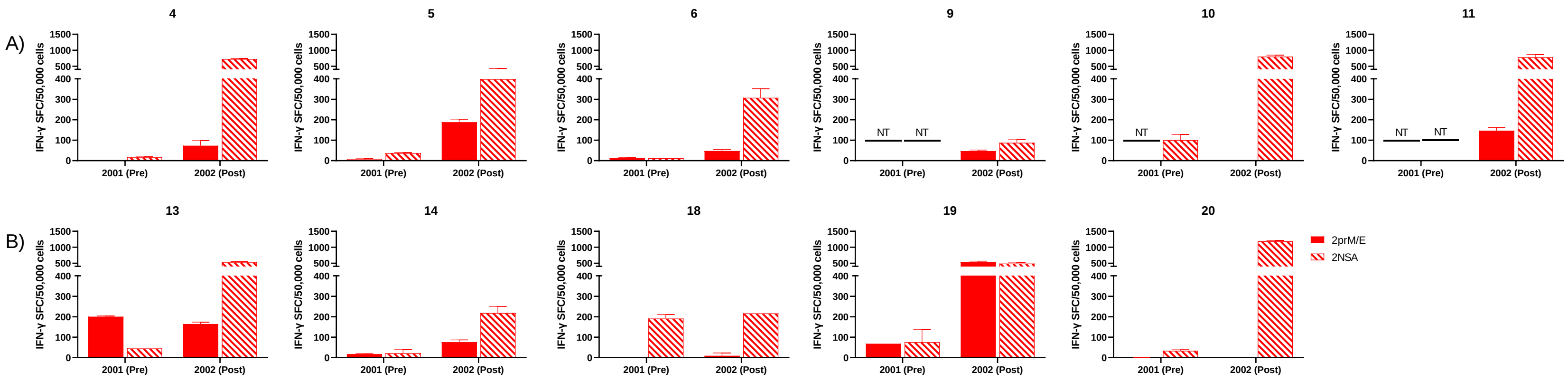

Figure S4. IFN-γ responses tested by cultured ELISPOT in individual subjects before and after a secondary DENV infection. DENV peptide-specific interferon γ (IFN-γ) spot-forming cells (SFC) were determined by cultured IFN-γ ELISPOT in PBMC collected before and after a secondary DENV-2 infection in subjects who were NH (A) and hDHF (B). PBMC were stimulated with peptide pools corresponding to prM and E proteins (prM/E) or NS1, NS3, and NS5 proteins (NSA) of the indicated DENV serotype. Bars represent mean with the standard error of the mean from duplicate wells. NT, not tested.

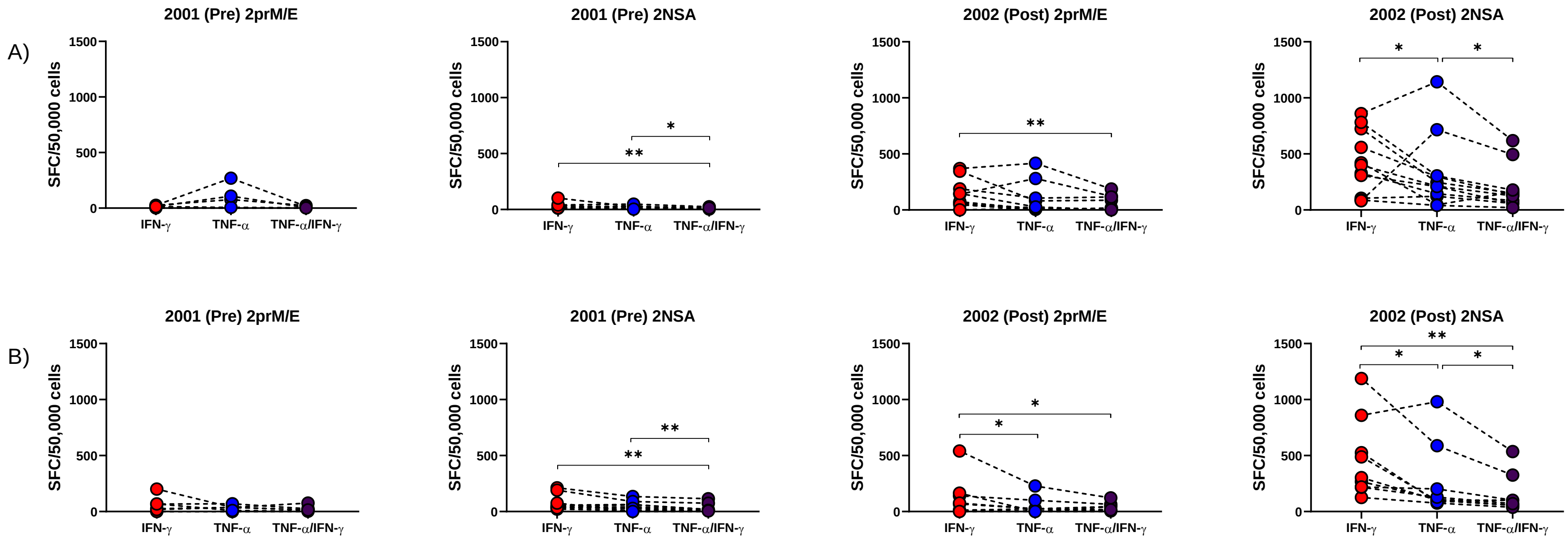

Figure S5. Dominant production of IFN-γ over TNF-α or IFN-γ/TNF-α by DENV-specific T cells. 2prM/E and 2NSA-specific specific IFN-γ, TNF-α and IFN-γ/TNF-α spot forming cells (SFC) were detected by cultured DC ELISPOT at 2001 and 2002 in NH (A) or hDHF (B) subjects. Circles represent the frequency of cytokine-producing cells in PBMC from each subject. Statistics were calculated by the non-parametric Wilcoxon matched-pairs signed-rank test. * $p < 0.05$, ** $p < 0.01$.

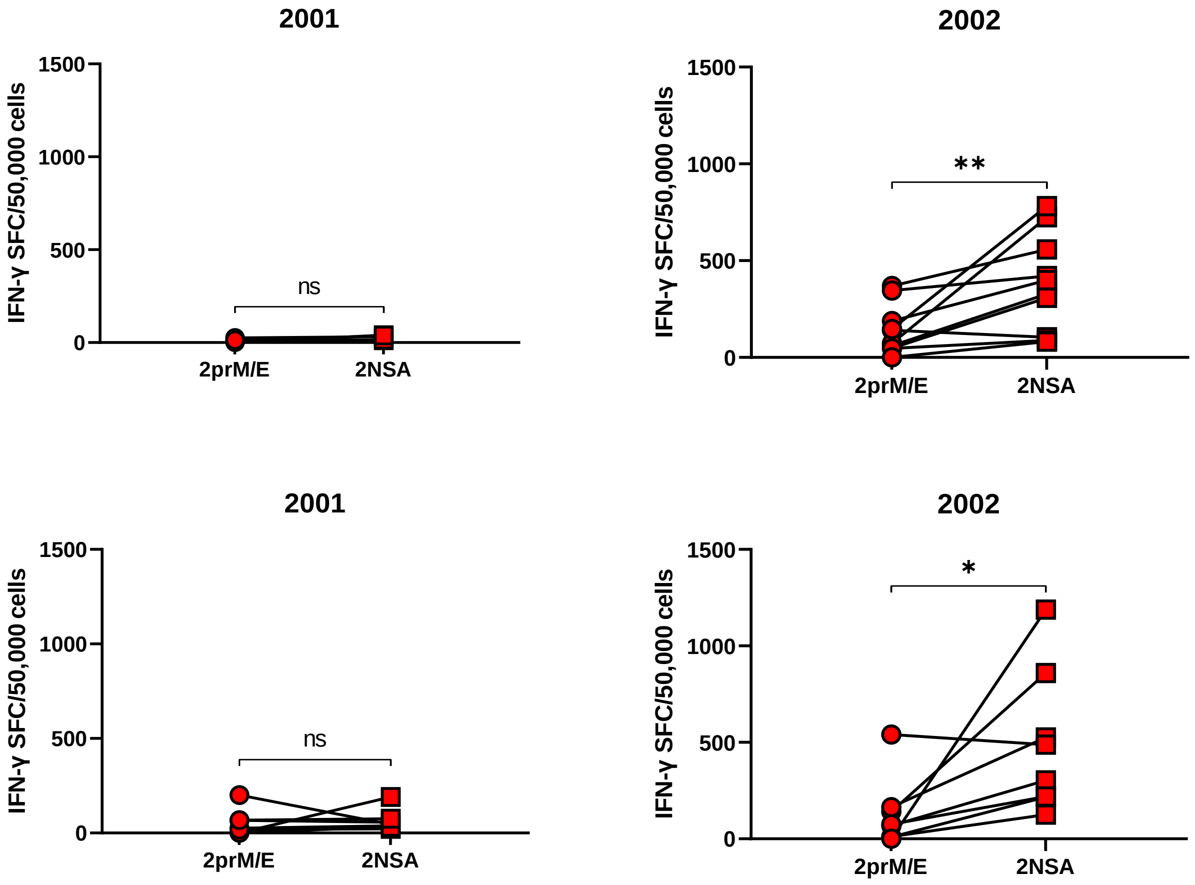

Figure S6. Preferential T cell activation by non-structural peptides from DENV-2. 2prM/E and 2NSA-specific IFN-γ spot forming cells (SFC) were detected by cultured ELISPOT after secondary infection in NH or hDHF subjects in 2001 and 2002. Symbols represent the frequency of cytokine-producing cells in PBMC from each subject. Statistics were calculated by the non-parametric Wilcoxon matched-pairs signed-rank test. * $p < 0.05$, ** $p < 0.01$.
